# A Local Experience of Antibiotic Lock Therapy as an Adjunctive Treatment for Central Venous Catheter-Related Bloodstream Infections in Pediatric Oncology and Hematology Patients

**DOI:** 10.3390/children11080983

**Published:** 2024-08-14

**Authors:** Elena de Frutos Porras, Elvira Cobo-Vázquez, Alicia Hernanz Lobo, María del Mar Santos Sebastián, Elia Pérez Fernández, Carmen Garrido Colino, Elena Cela, María Luisa Navarro Gómez

**Affiliations:** 1 Hospital Fundación de Alcorcón, C/Budapest 1, 28922 Madrid, Spain; elena.frutos@salud.madrid.org (E.d.F.P.); elvira.cobo@salud.madrid.org (E.C.-V.); eliapf@salud.madrid.org (E.P.F.); 2 Hospital Maternoinfantil Gregorio Marañón, c/O’Donnell 46, 28009 Madrid, Spain; alicia.hernanz@quironsalud.es (A.H.L.); mmar.santos@salud.madrid.org (M.d.M.S.S.); cgarridoc@salud.madrid.org (C.G.C.); elena.cela@salud.madrid.org (E.C.); 3 CIBER de Enfermedades Infecciosas, Instituto de Salud Carlos III, 28029 Madrid, Spain; 4 Unidad de Investigación Maternoinfantil Fundación Familia Alonso, Instituto de Investigación Sanitaria Gregorio Marañón, Universidad Complutense de Madrid, 28040 Madrid, Spain

**Keywords:** central venous catheter, antibiotic lock therapy, catheter-related bloodstream infection, pediatric, hematology, oncology

## Abstract

Background: One of the main drawbacks of tunneled central venous catheters (CVCs) is catheter-related bloodstream infections (CRBSIs). Antibiotic lock therapy (ALT) can be combined with systemic antibiotics to achieve catheter salvage. Our objectives are to describe cases of CRBSI and our experience with ALT in a pediatric oncology–hematology ward. Methods: a retrospective descriptive study of pediatric CRBSI cases in a Spanish oncology–hematology unit from 2007 to 2017 was conducted. We collected demographic, clinical, and microbiological data from all patients. Results: fifty-eight CRBSIs were diagnosed in thirty-nine patients; 72.9% of these patients were male, with a median age of 42.1 months. The main underlying diseases were leukemia/lymphoma (51.7%) and solid tumors (32.7%). Thirty-five (60.3%) CRBSIs were caused by Gram-positive cocci, of which 70.6% were coagulase-negative *Staphylococci*, and sixteen (27.6%) were caused by Gram-negative bacilli. We treated 41/58 (71%) cases with ALT. A total of 12/17 (71%) CVCs that were not treated with adjunctive ALT were removed, compared with 13/41 (32%) that were treated with ALT (relative risk (RR), 0.449; confidence interval (CI), 95%: 0.259–0.778, *p* = 0.004). Major reasons to remove the CVC in the CRBSI-ALT group were local insertion/pocket site infection (23%), persistent symptoms (23%), and infectious’ relapses (15%). Conclusions: ALT was shown to be an effective approach to keeping the CVC in place, with no added adverse effects.

## 1. Introduction

Tunneled central venous catheters (CVCs) are essential in the management of oncology–hematology patients [1,2,3,4]. These venous devices are used to infuse patients with most medications, chemotherapies, total parenteral nutrition, and blood components, and are also used to perform blood extractions to avoid repetitive venopunctions [3,4,5,6]. This CVC approach is of special relevance since it is not always feasible to obtain new venous access. Furthermore, CVCs also help prevent and minimize patient anxiety and pain related to these procedures.

On the other hand, the use of CVCs is not free from challenges, with bacteremia being one of their main drawbacks. Catheter-related bloodstream infections (CRBSIs) have a significant impact on morbidity and mortality due to their direct and indirect effects, such as delays in essential treatments in, for example, chemotherapy sessions [3,5]. Additionally, they prolong the length of hospital stay and the economic costs of patient care [3,7,8,9,10,11].

It has been estimated that CRBSIs in pediatric oncology–hematology patients have a mortality rate of 9.6% [12]. The main microorganisms described to be responsible for these infections are skin flora bacteria and skin commensals such as coagulase-negative *Staphylococci* (CoNS) or *Staphylococcus aureus*; other bacteria such as *Enterococcus* spp., *Escherichia coli*, and *Klebsiella* spp. are also common [1,13,14]. Some of the bacteria listed above can also create biofilms and corrode the catheter lumen, making treatment of these infections more challenging [11]. Fungal infections are also becoming of concern as CRBSI treatment and oncology patient survival improve.

CRBSI prevention using simple measures such as correct handwashing or site disinfection before any manipulation is indispensable [8].

While infection rates may vary, the rates in oncology–hematology patients have been reported to have an incidence from 1.4 to 1.9 instances per 1000 line-days [4]. In some cases, the CVC must be retired to facilitate recovery from infection [10]. In others, relapses in infection are caused as a result of the bacteria not being successfully eradicated despite the correct therapy being used. Moreover, difficulties obtaining venous access in children, the invasive characteristics of installing new CVCs, and CVCs’ high costs have motivated medical doctors to attempt catheter salvage whenever possible [3,4,5,6].

### Antibiotic Lock Therapy

Antibiotic lock therapy (ALT) is combined with systemic antibiotics in an attempt to achieve catheter salvage in the event of a CRBSI. This combination has been supported by different studies, but evidence of its effectiveness is scarce among pediatric populations. ALT implies the installation of a high antibiotic dose mixed with an anticoagulant to fill the CVC’s lumen for a specific amount of time (normally from 4 to 48 h, depending on the stability of the final compound). Its main purpose is to sterilize the lumen by reaching high local concentrations of the antibiotic. Antibiotics are chosen bearing in mind the pharmacodynamics and the causative bacteria.

The concentration that must be reached in the catheter’s lumen has to be 100 to 1000 times higher than the minimal inhibitory concentration. The anticoagulant (for example, sodium heparin 1000 UI/mL) helps obtain a stable mixture, which allows the antibiotic to better penetrate the biofilm and prevents the formation of clots. This type of treatment lasts from 7 to 14 days, considering the first day of negative culture as the first day of treatment.

Adjunctive ALT can be attempted when the patient is in a stable condition and the CRBSI is caused by vancomycin-sensitive CoNS, Gram-negative, or *Enterococci* bacteria [11]. ALT should not be attempted in cases of hemodynamic instability, sepsis, fungal infections, mycobacteria or *S. aureus* infections, or in the presence of complications such as endocarditis, septic emboli, or suppurative thrombophlebitis. In addition, if no clearance of blood cultures is observed after 72 h of adequate antibiotic therapy, the CVC should be removed [15,16,17,18,19,20,21,22,23,24].

The aim of this study is to describe the CRBSI cases in our oncology–hematology ward from January 2007 to September 2017. As secondary objectives, we look to describe our experience with ALT treatment in an attempt to accomplish catheter salvage and find traits that may predict successful salvage or the need for catheter removal.

## 2. Materials and Methods

### 2.1. Definitions

In our institution, the criteria for CRBSI diagnosis are based on the Infectious Diseases Society of America (IDSA)’s recommendations. Qualitative methods are employed based on the differential time to positivity standards [19].

A CRBSI is diagnosed when one of the following criteria are met:

- After obtaining a set of blood cultures, drawn at the same time and with an identical amount of blood; positivity in the CVC culture should occur at least 2 h before the percutaneous blood culture.
- A positive isolate in at least one peripheral blood culture and evidence of CVC colonization by the same microorganism. CVC colonization is proven when the tip or reservoir is cultured (catheter must be removed).

When no percutaneous blood culture is drawn:

- At least 2 blood cultures drawn from the CVC must have the same microorganism on 2 different occasions.

We defined severe neutropenia as a count of <500 neutrophils/mm^3^.

### 2.2. Methods

We performed a retrospective, descriptive study of CRBSI cases that occurred in our oncology–hematology unit of Gregorio Marañón University Hospital in Madrid (Spain), a university-teaching tertiary referral hospital from January 2007 to September 2017.

We looked at patient medical records for data on sex, age, underlying condition, date, and past medical history of bone marrow transplantation (BMT), past medical history of CRBSI, date of admission, past history of total parenteral nutrition, total white cell count, presence and length of severe neutropenia, date of CVC insertion, type of CVC, site of CVC insertion, date of CRBSI diagnosis, cultures obtained and their results/isolates, antibiotic regimen and regimen length, use of ALT as an adjunctive treatment (type of antibiotic used, duration), prognosis, relapses in infection, and the final outcome (Supplementary Materials).

### 2.3. Antibiotic Selection

After the suspicion or diagnosis of CRBSI, empirical antibiotic therapy (endovenous antibiotics with or without ALT) was always started, based, if possible, on previous isolations from the patient and always taking into account the patient’s general condition. As soon as microbiological confirmation was obtained, targeted antibiotic therapy was performed.

Regarding ALT, the most frequently used antibiotics were vancomycin for Gram-positive isolates and amikacin for Gram-negative ones. In the case of antibiotic resistance, once the sensitivity was confirmed, we switched to a different and more appropriate antibiotic. We have attached a small adaptation of the pamphlet and protocol currently used for more information regarding our empirical therapy and ALT.

### 2.4. Ethical Considerations

The study was approved by the Ethics Committee of Gregorio Marañón University Hospital. No informed consent was required due to the study’s retrospective design. All data were collected on an anonymized data set. All the patients admitted to the oncology–hematology ward with a CRBSI diagnosis were included in the study. Data were retrieved by reviewing medical records (paper charts from 2007 to 2013, and electronic ones from 2013 to 2017). The reviewed data were gathered anonymously, ensuring there was no possibility for the patients included in our study to be identified afterwards.

A standardized digital form was used to perform the data collection, and subsequently, the retrieved information was added to our database. Only the investigators of this project were allowed to access the database.

### 2.5. Statistical Analysis

The results of categorical variables are presented in percentages and frequencies, and median and interquartile ranges (IQR) are used to present the results from continuous variables. 

Categorical variables were analyzed using the Chi-square test or Fisher test, and for continuous variables, we used the Mann–Whitney U test.

We used modified Poisson regression [25] to estimate relative risks and its 95% confidence intervals. Statistical significance was established at *p* < 0.05. For the univariate analysis, we used the SPSS 17.00 (SPSS Inc., Chicago, IL, USA) and STATA 14 package for the multivariate analysis.

## 3. Results

We retrieved data on 58 CRBSIs among 39 patients admitted to our oncology–hematology ward between 2007 and 2017. The main characteristics of the CRBSIs and the patients are summarized in Table 1.

The diagnosis of CRBSI was performed using paired blood cultures in 51/58 cases (87.9%), conclusive tip culture in 1/58 (1.7%), tip culture and paired blood cultures in 5/58 (8.6%), and a local swab of purulent discharge in 1/58 (1.7%).

In our series, CoNS were the most frequent cause of CRBSIs, followed by Gram-negative bacilli and *Staphylococcus aureus*. The culture isolates and their frequencies are described in more detail in Table 2.

Out of 58 cases, 41 were managed using ALT with systemic antibiotics. In Table 3, the characteristics of the population managed with ALT are compared with those of the population who did not receive ALT as part of the therapeutic regimen.

The antibiotics and their concentrations used for lock therapy in our ward are found in Table 4. In all cases, the anticoagulant used was heparin (sodium heparin 1000 UI/mL). No adverse effects secondary to ALT were noted in the medical records.

The mean duration of the ALT was 11.24 days (standard deviation (SD): 5.713), the mean duration of the antibiotic regimen was 15.35 days (SD 7.441), and the antibiotic regimen with adjunctive ALT was 14.8 days (SD 6.005). There were no major differences in treatment duration compared to when ALT was added to the antibiotic treatment (*p* = 0.685).

Of the 41 cases managed with adjunctive ALT, 13 catheters needed to be removed, and in the 17 cases managed without ALT, 12 patients needed their catheters removed (relative risk (RR), 0.449; IC 95%: 0.259–0.778, *p* = 0.004). Univariate subanalysis among cases with an indication of ALT based on the IDSA guideline’s recommendations [19] showed a similar effect size, but it was not significant (RR: 0.531; IC 95%: 0.253–1.114, *p* = 0.094). In this population, a multivariate regression analysis was performed, and we found a significant effect of ALT treatment on catheter removal, with an RR of 0.339 (*p* = 0.033; IC 95%: 0.164–0.928), adjusted by age of the patient, type of underlying condition, severe neutropenia, and days from CVC insertion to infection.

A total of 25/58 catheters were removed. No fatal end-points secondary to CRBSI were retrieved.

In our case series, the reasons for catheter removal are described in more detail in Table 5 and will be discussed later.

We compared the general features of the catheters that were removed and those that were saved among the population with adjunctive ALT (Table 6). No statistically significant differences were found.

As CoNS were our most common isolate, we specifically examined that subpopulation to determine whether catheter salvage was more prevalent and compared the use of adjunctive ALT. The results were not significant (OR: 0.625; IC 9%: 0.046–8.432, *p* = 1).

We performed a multivariate analysis with the following variables: ALT, age of the patient, type of underlying condition, severe neutropenia, and days from CVC insertion to infection among the population with an indication of adjunctive ALT therapy based on the IDSA guideline’s recommendations [19]. We found significance for the variable ALT as adjunctive therapy with an RR of 0.339 (*p* = 0.033, IC 95%: 0.164–0.928) for catheter removal. The results are shown in Table 7.

## 4. Discussion

Consistent use of ALT as an adjunctive treatment for central venous catheter-related bloodstream infections in pediatric oncology and hematology patients to prevent catheter removal was found to be significant in our univariate and multivariate analyses. No adverse effects were described, which is congruent with other publications [1,11,12,23].

In our multivariate analysis, when we adjusted by age, severe neutropenia, underlying condition, and days from CVC insertion to infection, ALT was associated with fewer catheter removals among the population with ALT indication. The sample size limited the statistical analysis because possible adjustments according to different variables were limited. However, the positive effect that was identified cannot be ignored.

The sense of the results, when we look for the reasons for catheter removal (Table 6) in this case series (i.e., type of microorganism, local site infection, hemodynamic instability, and persistent signs and symptoms), must be interpreted with caution because they can be explained by the motives for removal themselves indistinctly from the use of ALT. As we can see, finding differences in the use of ALT and catheter removal is expected since both are closely related. The type of bacteria also determines the use and continuity of ALT and the consequent removal of the catheter. So, it is expected to observe those differences if we adhere to the IDSA guideline’s recommendations [19].

Similarly, a small, settled number of infectious relapses should be expected in the group of CRBSI patients treated with “ALT” versus the “no ALT group” if the catheter remains in place and the diagnosis of CRBSI is correctly made (with the catheter as the source of infection).

In our sample, we did not find infectious relapses in the group with “no adjunctive ALT”, which leads us to believe that our CRBSIs were correctly diagnosed. Moreover, having only two relapses in the ALT group does not allow us to perform a study with enough potency to identify the relation between ALT and infectious relapses, and, as explained earlier, two can be expected under “normal” circumstances. We could not conduct a study to find a specific trait predisposing patients to relapse in the “ALT group” since an *n* of two offers a poor perspective for finding any valid predisposing characteristics.

The same can be said of the single case we have in the category of hemodynamic instability since it is a direct criterion for catheter removal and, hence, salvage with ALT was not attempted.

The diagnosis of pocket site infections is not always immediately feasible, especially among oncology patients. Signs and symptoms can appear with a certain delay, which means treatment with ALT is typically started until it becomes clearer that the source of infection is the pocket or the site of the catheter. This happened in three of our patients. While it is true that it is a catheter infection, the cause does not remain in the lumen where the ALT dwells. It is expected that the ALT will fail under these particular situations, so comparing the groups will be of no help. In the subgroup of persistent signs and symptoms, the cases were equally distributed, so the significance of the results does not correlate with this category and no assumptions can be made.

Unfortunately, as we can see in Table 6, no distinctive features among the retired or saved catheters treated with adjunctive ALT were found in our comparisons. A larger sample could likely help us find an answer to this relevant question.

The most common organisms isolated from the cultures were Gram-positive cocci, with a predominance of CoNS. This finding is in agreement with the results described in a vast majority of the literature that was reviewed [1,2,3,5,6,8,9,10,12,14,19].

In the univariate analysis, the beneficial effect of ALT could not be found when we performed a subgroup analysis while specifically looking for its effects among cases with CoNS as the CRBSI’s etiology. This was also the case when we grouped and compared Gram-positive cocci, Gram-negative bacilli, fungi, and polymicrobial etiologies. In one publication with Gram-negative bacilli as the predominant source of CRBSI, the constant use of ALT with systemic antibiotics yielded a positive impact with fewer CVCs removed; however, no subanalysis was performed [11]. This leads us to conclude that ALT may be more efficacious in response to specific bacteria instead of general, widespread use.

As we mentioned previously, CoNS were the predominant etiologies and, thus, played an important role in our results. The sample size in our research was small and limited us when exclusively studying CoNS CRBSIs; regardless, its influence cannot be overlooked, even though the subanalysis of the relation between CoNS’s etiology, ALT, and catheter salvage was not significant. Due to the positive results in the statistical analysis when looking for an overall effect of ALT, we would like to think that ALT may have an effect in treating CoNS.

There are more data regarding the use of prophylactic ALT to prevent/treat CVC colonization, where the most frequently implicated bacteria are CoNS. The use of vancomycin in these patients’ lock solutions was enough to eliminate the biofilm and subsequently remove infections. As previously stated by other authors, these results should be interpreted cautiously because of the variability among the studies, and no strong assumptions should be made [12]. In our study, we can see from the comparison of saved versus removed catheters treated with adjunctive ALT that vancomycin is more prevalent among cases with saved catheters than in those where the catheter was removed, as well as in the treatment of CoNS, but with no statistical significance in either case. We would like to think there is potential for those traits based on what has been reported previously. An adequate use of ALT may be an effective treatment for CoNS biofilms and salvaging CVCs, even if current evidence is scarce and not always consistent/robust. The existing evidence points out that ALT using systemic antibiotics could be an effective treatment for CoNS CRBSIs. However, this has not yet been proven, so bigger and wider case series need to be designed in order to shed more light on the use of ALT or a specific antibiotic, such as vancomycin, and its specific effects on different microorganisms, as has been similarly pointed out in other publications. [1,11,12,23].

While other studies have evaluated the use of ethanol lock therapy as a treatment and secondary prophylaxis of CRBSIs, evidence suggests that it increases the risk of major adverse events such as CVC occlusion and minor ones such as pruritus or chest pain and that it does not appear to be effective [26,27]. Its effectiveness has only been demonstrated when used in hemodialysis catheters in adult patients [28]. Therefore, ethanol lock therapy cannot be recommended in pediatric oncology or hematology patients [26].

Mucosal barrier injury–laboratory-confirmed bloodstream infections (MBI-LCBIs), a concept introduced by the Centers for Disease Control and Prevention (CDC), are not routinely used in our oncology–hematology unit [29]. Few studies have compared the acuity of IDSA criteria versus those proposed by the CDC in the pediatric population to identify the true bacteremia related to CVC. Chaftary et al. pointed out in their paper in 2016 that excluding all pediatric patients with potential mucosal barrier injuries, such as oncology patients, from the possibility of also having CRBSIs could lead clinicians to underdiagnose CVC issues as life-threatening problems [30]. We should not forget that these vulnerable patients are prone to developing both mucosal barrier injuries and CRBSIs. Clinicians are encouraged to diagnose CRBSI using laboratory findings and to use the IDSA criteria to rule out the influence of MBIs in this type of patient. Making a more accurate CRBSI diagnosis will help to avoid unnecessary CVC removal or, on the contrary, futile or excessive CVC manipulation [31]. We believe that not using the MBI concept in our series was not a relevant issue since our CRBSIs are mostly laboratory proven.

There may be an exception to this statement if we look into the case of a CRBSI caused by *S. aureus* in the ALT group, in which against all odds, the CVC was saved. This 2-year-old patient with Ewing sarcoma in 2014 started with a fever, and the first set of hemocultures came back positive for *S. aureus*. Therapy was initially started with vancomycin as ALT, and the next set of cultures showed a clearing of the hemoculture obtained from the CVC in less than 24 h. The peripheral sample did not show such an early clearing. The patient remained stable, with no progression of the symptoms, and the team decided to maintain the CVC. After finishing his antibiotic course, no secondary infectious relapses caused by *S. aureus* were noted. We think that at that stage, with those ambiguous results and considering the good condition of the patient, the medical team decided not to retrieve the catheter and went for a 10-day course of ALT treatment and a full course of IV antibiotics. The team diagnosed it as CRBSI in 2014, but when retrospectively looking into it, we think it may be an MBCI- LCBIs more than a CRBSI and that there is a possibility the ALT could have prevented the colonization of the CVC. We did not exclude this case because we believe we are presenting a reflection of clinicians’ daily practice in a pediatric hematology–oncology ward. To us, the sample is more representative of what a pediatrician can face, and, hence, data is more valuable because they are more relatable to the specialist facing these patients. It also gives a chance to invite the reader to think of other possible benefits of ALT beyond treatment.

In our case series, patients with CRBSIs were diagnosed based on the IDSA criteria [19]. Achieving these diagnoses with laboratory confirmation is one of the main strengths of this study. In our hospital, the oncology–hematology unit consults an infectious diseases unit each time a CRBSI is suspected. This helps to ensure appropriate adherence to protocols and guidelines, the importance of which is pointed out by Hecht et al. [31], and this practice can explain why our CRBSIs are laboratory proven.

In the pediatric population, set blood cultures are not always feasible and are not an uncommon feature in CRBSIs, especially if the patient has a chronic underlying condition. Additionally, in chronically ill pediatric patients, healthcare providers look to limit the suffering caused by repetitive blood extractions [3].

There are limitations to our study. We advise the lector to interpret the data with caution because of the risk of some compromise of the external validity of our study due to the small size of our sample and the number of adjusted variables in our multivariate model.

Also, retrospective designs could present selection and information bias that we hope were minimized by using a CRBSI laboratory-proven and predefined form to collect the data.

Overall, we insist that our study adds to the current literature an exploratory value and hopes to incite other clinicians to investigate the issues we depict. We believe, based on the different elements presented in our discussion, that there is something genuine hidden between the data and some clues as to what ALT can perform.

## 5. Conclusions

The use of ALT as an adjunctive treatment for CRBSIs may help to reduce the risk of CVC removal in pediatric oncology–hematology patients. More studies are needed to clarify the role of ALT in CRBSs with CoNS infections, as well as in other microorganisms such as Gram-negative bacteria, and its potential to avoid catheter removal in these patients.

## Figures and Tables

**Table 1:** Main characteristics of the 58 CRBSIs and the patients.

Characteristics	Values
Age, months, median (IQR)	42.1 (22.8–91.83)
Male, n (%)	42 (72.4)
Underlying condition, n (%)	
Hematologic tumor	30 (51.7)
leukemia	26 (44.8)
lymphoma	4 (6.9)
Solid tumor	19 (32.7)
brain tumor	1 (1.7)
non-brain solid tumor	18 (31)
Other hematologic conditions	9 (15.5)
sickle cell disease	4 (6.9)
beta-thalassemia major	4 (6.9)
Langerhans histiocytosis	1 (1.7)
WBC cells/mm^3^ median (IQR)	2400 (400–7000)
Severe neutropenia, n (%)	
yes	21 (36.2)
no	37 (63.8)
Total parenteral nutrition, n (%)	3 (5.2)
Type of catheter, n (%)	
Port-a-Cath ^®^	51 (87.9)
Hickman ^®^	7 (12.1)
Catheter insertion site, n (%)	
subclavian	15 (25.9)
jugular	8 (13.8)
femoral	1 (1.7)
superior cava vein	1 (1.7)
missing data	33 (56.9)
Days from CVC insertion to infection, median (IQR)	192 (67.5–545.5)

CRBSIs: catheter-related bloodstream infections. IQR: interquartile range. WBC: white blood cells. CVC: central venous catheter. Severe neutropenia: <500 cells/mm^3^. N: 58 cases.

**Table 2:** Culture isolates in patients managed with and without ALT.

Culture Isolates	Frequency (% of the Total)	ALT in Therapeutic Management (Salvage Achieved)	No ALT in Therapeutic Management (Salvage Achieved)
Gram-positive cocci	34 (58.6)		
CoNS	24	21 (16)	3 (2)
*S. aureus*	5	2 (1)	3 (0)
*Enterococci*	1	-	1 (1)
*Streptococci*	4	2 (2)	2 (2)
Gram-positive bacilli	1 (1.7)		
*Corynebacterium* spp.	1	1 (1)	-
Gram-negative bacilli	18 (31.1)	13 (6)	5 (0)
*E. coli*	4	4 (3)	-
*ESBL-producing E. coli*	1	1 (0)	-
*Klebsiella* spp.	2	1 (0)	1 (0)
*Enterobacter cloacae*	4	4 (1)	-
*Enterobacter asburiae*	1	1 (0)	-
*Pseudomonas* spp.	1	-	1 (0)
*Serratia marcescens*	1	-	1 (0)
*Burkholderia* spp.	3	1 (1)	2 (0)
*Acinetobacter* spp.	1	1 (1)	-
*Candida* spp.	2 (3.4)	-	2 (0)
Polymicrobial	3 (5.2)	2 (2)	1 (0)
Total	58 (100)	41 (28)	17 (5)

ALT: Antibiotic lock therapy. CoNS: *Coagulase-negative staphylococci.* ESBL: Extended spectrum beta-lactamase.

**Table 3:** Characteristics of patients managed with and without ALT.

Characteristics of Patients	Without ALT (n = 17)	With ALT (n = 41)	*p* Value
Age, months, median (IQR)	57.63 (27.45–98.9)	41.6 (22.67–139.37)	0.844
Male, n (%)	11 (64.7)	31 (75.6)	0.520
Underlying condition, n (%)			0.313
Hematologic tumor	7 (41.2)	23 (56.1)	
Solid tumor	6 (35.3)	13 (31.7)	
Other hematologic conditions	4 (23.5)	5 (12.2)	
sickle cell disease	3	1	
beta-thalassemia major	1	3	
Langerhans histiocytosis	0	1	
WBC cells/mm^3^ median (IQR)	1900 (200–6550)	2900 (600–700)	0.261
Severe neutropenia, n (%)			0.268
Yes	8 (47.1)	13 (31.7)	
Parenteral nutrition, n	2 (11.8)	1 (2.4)	0.203
Type of catheter, n			0.178
Port-a-Cath ^®^	13 (76.5)	38 (92.7)	
Hickman ^®^	4 (23.5)	3 (7.3)	
Catheter insertion site, n (%)			0.583
subclavian	4 (57.1)	11 (61.1)	
jugular	2 (28.6)	6 (33.3)	
femoral	1 (14.3)	0	
superior cava vein	0	1 (5.6)	
Days from CVC insertion to infection, median (IQR)	159.5 (21.75–331)	229 (98.5–747)	0.108
Gram-positive cocci	9	25	0.260
Gram-positive bacilli	0	1	
Gram-negative bacilli	5	13	
Fungi	2	0	
Polymicrobial	1	2	

ALT: Antibiotic lock therapy. CVC: Central venous catheter. IQR: Interquartile range. WBC: White blood cells. Severe neutropenia: <500 cells/mm^3^. N: 58 cases.

**Table 4:** Antibiotics used for lock therapy in our ward.

Antibiotics	Frequency n, (%)
Vancomycin 5 mg/mL	26 (63.4)
Amikacin 5 mg/mL	9 (22)
Ciprofloxacin 1 mg/mL	3 (7.3)
Gentamicin 5 mg/mL	2 (4.9)
Cefazolin 5 mg/mL	1 (2.4)
Total	41 (100)

**Table 5:** Reasons for CVC removal in patients with and without ALT.

Reasons for CVC Removal	No Adjunctive ALT	Adjunctive ALT	Total
Type of microorganism	7	1	8
Infectious Relapses	0	2	2
Local site infection	1	3	4
Hemodynamic instability	1	0	1
Persistent signs and symptoms	3	3	6
No data	0	4	4
Total	12	13	25

CVC: Central venous catheter. ALT: Antibiotic lock therapy.

**Table 6:** General features of patients with adjunctive ALT with and without removal of the CVC.

General Features	CVC Not Removed (n = 28)	CVC Removed (n = 13)	*p* Value
Age, months, median (IQR)	40 (23.4–215.8)	31.8 (21.8–58.4)	0.272
Male, n (%)	22 (78.6%)	4 (30.7%)	0.698
Underlying condition, n (%)			0.305
Hematologic tumor			
leukemia	12	9	
lymphoma	2	0	
Solid tumor	10	3	
Other hematologic conditions			
sickle cell disease	1	0	
beta-thalassemia major	3	0	
Langerhans histiocytosis	0	1	
No BMT/ with BMT, n (%)	19 (86.4)/3 (13.6)	13 (100)/0 (0)	0.279
WBC cells/mm3 median (IQR)	2650 (573–5625)	3000 (600–10,700)	0.612
Severe neutropenia, n (%)			0.493
no	18 (64.3)	10 (43.5)	
yes	10 (35.7)	3 (56.5)	
Total parenteral nutrition, n (%)	1 (3.5)	0 (0)	1
Type of catheter, n (%)			0.539
Port-a-Cath ^®^	25 (89.3)	13 (100)	
Hickman ^®^	3 (10.7)	0	
Catheter insertion site, n (%)			1
subclavian	7 (58.3)	4 (66.7)	
jugular	4 (33.4)	2 (33.4)	
femoral	0	0	
superior cava vein	1 (8.3)	0	
not recorded/missing data	0	0	
Type of ALT			0.0336
vancomycin	20 (71.4)	6 (45.1)	
amikacin	4 (14.3)	5 (38.5)	
ciprofloxacin	2 (7.1)	1 (8.2)	
cefazolin	1 (3.6)	0	
gentamicin	1 (3.6)	1 (8.2)	
ESBL, n (%)	0	1 (8.2)	0.325

CVC: Central venous catheter. IQR: Interquartile range. BMT: Bone marrow transplantation. WBC: White blood cells. ALT: Antibiotic lock therapy. ESBL: Extended spectrum beta-lactamase.

**Table 7:** Multivariate analysis.

	RR (CI 95%)	*p* Value
Antibiotic lock therapy	0.390 (0.164–0.928)	0.033
Age	1.00 (1.00–1.00)	0.195
Type of underlying condition		
all hematologic conditions	0.611 (0.226–1.654)	0.332
solid tumor	0.401 (0.152–1.054)	0.064
Severe neutropenia	0.549 (0.206–1.468)	0.232
Time from CVC insertion to infection (days)	1.000 (0.999–1.001)	0.457

RR: Relative risk.

## Data Availability

The raw data supporting the conclusions of this article will be made available by the authors on request.

## Supplementary Materials

The following supporting information can be downloaded at: https://www.mdpi.com/article/10.3390/children11080983/s1, Annex S1: Adapted and modified from “Approach to the pediatric febrile patient with neutropenia or stem cell transplantation”. Table S1: High risk patient; Table S2: Antibiotherapy. Annex S2: Adaption of “Recommendations for prevention and treatment of catheter-related bloodstream infections”. Table S3: Preparation of antibiotic lock solution; Table S4: Empiric antibiotic therapy (choose based on clinical suspicion); Table S5: targeted antibiotic therapy.
