# Supplementary material for: A Local Experience of Antibiotic Lock Therapy as an Adjunctive Treatment for Central Venous Catheter-Related Bloodstream Infections in Pediatric Oncology and Hematology Patients
Source: Children (Basel). 2024 Aug 14;11(8):983. doi: 10.3390/children11080983 (PMC11352447; doi:10.3390/children11080983)
Supplement: Supplementary file 1 [file children-11-00983-s001.zip › children-3029181-supplementary.pdf]

High risk patient (Table S1)

Admission and:

- Piperacilin/tazobactam 320 mg/kg/day, qid, or
- Meropemem 60mg/kg/day, tid, or
- Cefepime 150 mg/kg/day, tid

If suspicion of CRBSI consider adjunctive antibiotic lock therapy

Associate vancomycin/teicoplanin or an aminoglycoside if criteria are met (see Table S2)

Asses daily + aim for targeted therapy

At 96 hours of treatment:

Patient stable, afebrile

Readjust therapy

Patient stable but febrile

Reassess clinical-physical condition and diagnosis

No further changes in therapy

Patient unstable or signs of progression

Check:

- coverage of resistant G+ or G-
- coverage of fungi
- coverage of anaerobia bacteria

Reassess laboratory test+ imaging+ readjust empirical therapy.

Evaluate the risk of **invasive fungal infection**

Start investigations + antifungal therapy

- Consider asking for hemocultures, blood samples, urine, bronchial aspirate, tissue biopsies, galactomannan, (1,3)-β-d-glucan (BDG), imaging (abdominal US, CT scan,) ,...

Annex S1. Adapted and modified from " Approach to the pediatric febrile patient with neutropenia or stem cell transplantation". Clinical guideline. E. Cobo Vazquez. J Saavedra Lozano. feb 2019, revised Feb 2022. H. Universitario Gregorio Marañón.

Table S1. High risk patient

Patient	Condition	Laboratory findings
Fever in patient already admitted<br>Comorbidities:<br>- Hemodynamically unstable<br>- Severe mucositis (stage III-IV)<br>- Altered mental status<br>- Hypoxemia, shortness of breath, chronic lung disease, lung infiltrates<br>- CRBSI suspicion<br>Personal background<br>- Difficulties to access care (>1hrs distance)<br>- Difficulties with home care, risk of poor compliance to the treatments	High risk condition:<br>- acute myeloid leukemia, high risk acute lymphoblastic leukemia, Burkitt lymphoma, myelodysplastic syndrome, stage IV neuroblastoma,<br>- allogenic stem cell transplant (specially first year, and under immunosuppressant regimentn engraftment),<br>- autologous stem cell transplant (specially <31 days)<br>- condition relapse or difficulties to control the disease<br>- graft versus host disease<br>- antibiotics in the last week<br>- IFI en the past 6 months	- Severe and prolonged neutropenia (<500/mm3, >7-10 days)<br>- Monocytes <100/mm3<br>- CrCl< 30 or hepatic insufficiency (ALT, AST x5)<br>- PCR ≥9 mg/dL<br>- PCT ≥ 2 ng/mL

Table S2. Antibiotherapy.

Piperacilin/tazobactam (P/T)*	Cefepime	Meropemem
Patient with stable condition<br>No colonization by resistant bacteria<br>No previous infection by resistant bacteria	Patient with stable condition, presence of risk factors for IFI**<br>No history of colonization nor infection by resistant bacteria	Patient with unstable condition<br>Colonization/infection by resistant microorganisms

*P/T is the first choice antibiotic except in patients with high risk for IFI (it interferes with galactomannan)
** ** acute myeloid leukemia, high-risk acute lymphoblastic leukemia, Burkitt lymphoma, myelodysplastic syndrome, stage IV neuroblastoma, allogeneic stem cell transplant (especially first year, and under immunosuppressant regiment), autologous stem cell transplant (specially <31 days).

Amikacin 15-20 mg/kg/day, ask for blood levels if treatment for >72 hrs

Consider adding amikacin to the empiric regimen if:

- unstable condition
- Past history of colonization/infection by resistant bacteria
- Consider if recent history of carbapenems

Vancomycin (45-60 mg/kg/day, tid-qid), **Teicoplanin** (10 mg/kg/dose every 12 hours the first 3 doses, then every 24 hrs.)

- Patient hemodynamically unstable, suspicion of septic shock, severe sepsis. Consider alternative therapy if pneumonia is a concern.
- High incidence of SARM (not our current situation)
- Past history of colonization/infection caused by SARM or resistant streptococci
- Blood cultures with gram positives in the preliminary result/preliminary identifications
- Severe mucositis (stage III-IV) or chemotherapy that might cause it (fludarabine or high dosage of ARA-C). It should be reflected in the patient's current history
- High suspicion of CRBS or fever starts after CVC manipulation
- Pocket site infections or cellulitis

Terapeutic management:

Indications for maintainig the catheter:

- Reevaluate everyday if there is need for maintaining the catheter
- Try to maintain indispensable catheters when there is a no complicated CRBSI nor signs of local infection.
- In such cases treat with systemic antibiotics + antibiotic lock therapy for 7-14 days
  - Antibiotic lock (ALT): doublechek the catheter functionality. Lock every lumen a minimum of 2 hrs. and rotate the solution between the different lumen. (check Table S3 for the solution composition)
  - Systemic therapy:
    - empiric (Table S4)
    - Targeted therapy (Table S5.)

Indications for catheter removal:

- Check if the catheter is needed, or if any of the following endpoints is met:
  - Hemodynamic instability
  - Suppurative thrombophlebitis
  - Infectious endocarditis
  - Persistent bacteremia (>72 hrs. of treatment)
  - Infections caused by difficult to eradicate microorganism (*S.aureus, P. aeruginosa, mycobacteria*, fungi, etc.)

Treatment duration

1st day of treatment = first day of negative cultures

- CoNs: 5-7 days
- Gram negative bacilli: 10-14 days
- *S.aureus*: 14 days
- *Candida spp:* 14 days.

Table S3: preparation of antibiotic lock solution

			Final solution		
**Antibiotic**	**Presentation**	**Dilution**	**Volume to retrieve**	**0.9% SF or GS 5%**	**Heparin sodium 1000UI/mL vol.**
**Vancomycin** 500 mg	powder	+10 ml	5 mL	50 mL	3 ml
**Cefazolin** 1 gr	powder	+ 4 ml sterile water for injection	1 mL	50 mL	3 mL
**Ciprofloxacin** 2mg/mL	100 ml perfusion bag	NO	25 mL	50 mL	-
**Amikacin** 250 mg/mL	2 mL injection vial	NO	1 mL	50 mL	3mL

Table S4: Empiric antibiotic therapy (choose based on clinical suspicion)

Microorganism	Election	Indications
Gram +	Vancomycin/Daptomycin	Always cover SARM, SCN-MR
Gram -	Based on latest epidemiolgical data	Cover multi-R in neutropenic, severe patients, or Multi-R colonization
Fungi	Candine/fluconazol<br>(adjust based in epdemiolocal data)	Risk factors: neutropenia, transplantation,<br>Femoral access<br>Parenteal nutrition<br>Previous use of broad spectrum antibiotics<br>Personal hystory of candida colonization

Table S5: targeted antibiotic therapy

Microorganism	First choice	alternatives
S. epidermidis	Vancomycin	Daptomycin/linezolid
**SAMS**	Cloxacilin	Cefazolin
**SARM**	Vancomycin	Daptomycin/linezolid
E. faecalis	Ampicilin+/- aminoglycoside	Vancomycin
***Enterococcus* ampi-R**	Vancomycin +/- aminoglycoside	Linezolid/Daptomycin
***Enterococcus* Vanc-R**	Linezolid/daptomycin	
P. aeruginosa	Antipseudomonal beta-lactam agent	quinolone
**Enterobacteria**	Beta-lactam	quinolone
S.maltophila	TMP-sulphamethoxazole	
***Candida spp* fluco S**	fluconazole	Anphotericin B
Candida spp **fluco R**	Equinocandines	

Annex S2.: Adaption of ·"Recommendations for prevention and treatment of catheter-related bloodstream infections". Grupo de estudio de la infección asociada a dispositivos intravascular. Comité de infecciones, higiene y política de antibióticos. H. Universitario Gregorio Marañón
